# Transition in tobacco use stages and its related factors in a longitudinal study

**DOI:** 10.1186/s12199-018-0728-x

**Published:** 2018-08-18

**Authors:** Ahmad Khosravi, Mohammad Hassan Emamian, Hassan Hashemi, Akbar Fotouhi

**Affiliations:** 1 Center for Health Related Social and Behavioral Sciences Research, Shahroud University of Medical Sciences, Shahroud, Iran; 2 Department of Epidemiology, School of Public Health, Shahroud University of Medical Sciences, Shahroud, Iran; 3 Noor Ophthalmology Research Center, Noor Eye Hospital, Tehran, Iran; 4 Department of Epidemiology and Biostatistics, School of Public Health, Tehran University of Medical Sciences, PO Box: 14155-6446, Tehran, Iran

**Keywords:** Smoking, Tobacco use, Transition, Longitudinal studies, Iran

## Abstract

**Objectives:**

Considering the increase in the non-communicable diseases associated with tobacco use in recent decades in Iran, it is necessary to have a general view of the current condition. This study aimed to identify factors associated with tobacco use and to estimate the probability of a 5-year transition in the stages of tobacco use in an adult population.

**Methods:**

In this study, 5190 people in the 40–64-year-old population of Shahroud (North East of Iran) were interviewed in 2009 and 2014 on tobacco smoking. The association of independent variables with tobacco smoking was evaluated using the population-averaged logit model. We calculated smoking transition probabilities from non-smoking to current smoking and past-smoking stages during a 5-year span.

**Results:**

The prevalence of current tobacco smoking in 40–69-years age group was 11.1% (95% CI 10.3–12.0), 1% among women (95% CI 0.8–1.3) and 25.6% among men (95% CI 23.7–27.6). During this 5-year period, the probability of transition of a non-smoker to an overall current tobacco smoker was 2.3%. Meanwhile, 18.5% of the overall current tobacco smokers had changed into past smokers. Unemployed (OR = 2), male gender (OR = 53.9), widow/widowers (OR = 5.4), divorces (OR = 3.3), and high economic status (OR = 1.2) are associated to tobacco smoking.

**Conclusions:**

Compared with the other studies, the prevalence of tobacco use in this population is low but transition rate of non-smokers into current smokers or past smokers is high. Conducting interventions on determinants of starting and quitting smoking and education and awareness raising on the risk and harms of smoking seems necessary.

## Introduction

Tobacco smoking, after hypertension, along with high blood sugar level, overweight, and a sedentary lifestyle, is among the main risk factors of death in the world. Almost 9% of deaths are attributable to smoking and 13% to hypertension [1]. These risk factors are the most important causes of chronic diseases such as heart diseases, cancers, and diabetes [1]. As the World Health Organization (WHO) reports, tobacco use is increasing in countries with low and middle income and in high-income and developed countries, it is slowly declining with a constant slope [1, 2]. The rising trend of tobacco use in poor and middle-income countries will lead to increased deaths from smoking-related diseases in the coming years [1]. Among the middle-aged persons, tobacco use is estimated to be the most important risk factor for premature deaths in men in 2010–2025 [3]. Tobacco use in Western Europe decreased by 26% from 1990 to 2009, whereas in Africa the Middle East, it has increased by 57% during the same time [2]. In a study of smoking-related deaths in the USA in a 50-year period, men and women smokers’ risk of death from all causes was at 2.8 and 2.76 times higher than non-smokers’ risk, respectively [4]. Passive smoking, or environmental tobacco smoke, has been found to be associated with respiratory symptoms, asthma, a small but significant impairment of lung function, and increased bronchial responsiveness [5]. In another study, passive smoking was associated with an excess risk of coronary heart disease [6].

According to WHO estimates for 2009 in Iran, the prevalence of smoking any tobacco product in the population over 15 years was 26% among men and 2% among women, while according to the same report, the global prevalence of smoking any tobacco product was 36% among men and 8% among women, and the prevalence of tobacco in the Middle East countries for the two groups was 33% and 4%, respectively [7]. In the Persian Gulf Healthy Heart Study, smoking prevalence among people over 20 years in Bushehr, south of Iran, in the first phase was 11.3% and in the second phase was 7.5%. While the prevalence of hookah (water pipe) in the first and second phases was 17.9% and 12.9%, which reflects the trend of declining consumption in a cohort of patients in a 6-year period, although only 49.6% of people participated in the second phase of the study. This missing to follow-up can influence the estimate of smoking cigarette and water pipe [8]. Another study in Tehran reported the prevalence of smoking in the population over 15 years old to be 11.9% [9]. The results of the national survey of risk factors of non-communicable diseases (SuRFNCD-2007) on 5287 people between 15 and 64 years old from all over the country showed that the prevalence of tobacco consumption was 14.8% (95% CI 12.2 to 17.9) and the prevalence of smoking was 12.5% [10]. In Tehran Glucose and Lipid Study (TGLS), the prevalence of smoking (daily and recreational) was 24.5% among men older than 15 years old and 2.8% among women [11]. In a review study by Moosazadeh et al., which aimed to examine the prevalence of tobacco use, the prevalence of smoking in different parts of Iran was between 7 and 30% and these differences may result from the differences in the definition of smoking and in the age ranges of the participants [12]. Studies conducted with Iranian adolescents show an increase in tobacco use over the recent, which can lead to an increase in the prevalence of smoking in older ages and consequently can lead to more smoking-related diseases and deaths [13].

Most of the studies on tobacco consumption have been cross-sectional, and in the Persian Gulf Healthy Heart Study, the data of the two phases were analyzed independent of each other and one of the weaknesses of the study was that in the second phase, about half of the participants left the study. Another cohort study on the pattern of smoking consumption and transition among young adults was conducted by Mohammadpoor et al. in 2010 and 2011. In this study, 5197 high school students were followed for 1 year. In this study, over a year, 10.1% and 1.7% of non-smokers were transmitted to experimenter and regular smoker groups, respectively [14]. In the present study, the data of the two phases of Shahroud Eye Cohort Study (ShECS) were examined in a longitudinal analysis. Given that we examined smoking among the sample within a 5-year period, and over time for various reasons, such as disease and health problems, people may quit smoking, it seems necessary to examine the transition pattern in different stages of smoking. Given the epidemiological transition in the country and increase in non-communicable diseases associated with smoking in recent decades in the country, it is necessary to have a general view of the current condition. This study aimed to estimate the prevalence of tobacco use, to identify factors associated with tobacco use and quitting, and to estimate the probability of the 5-year transition in the stages of tobacco use in the adult population of Iran.

## Method

Data on the tobacco smoking stage transition and risk factors were obtained from Shahroud Eye Cohort Study (ShECS). The methodology of this study has been presented in detail previously [15]; here, it is briefly described. In the study, using cluster stratified sampling, 300 clusters in nine strata were randomly selected from Shahroud (a city in the North East of Iran). The strata were health care centers. At least twenty 40- to 64-year-old individuals were selected from each cluster to participate in the study. From 6311 individuals who were selected for this study, 5190 participants (82%) took part in the first phase of the study in 2009. After 5 years in 2014, the same people were examined and interviewed again in the second phase of the study. In the second phase of the study, 453 people (8.7%) did not participate in the study. Following the matter up, it was known that 108 people among these had died during the 5-year period. Therefore, 4737 people participated in the second phase and the participation rate was 93.2% of the survivors from the first phase.

Upon enrollment at both phases, each participant signed a written informed consent, had an interview, and was then examined. In the interviews, demographic factors such as employment status, smoking status, medical history, and ophthalmology records were taken into account. The study protocol was reviewed and approved at both phases by the ethics committee of Shahroud University of Medical Sciences.

In both phases of the study, questions were asked about the status of smoking, frequency of consumption, type (cigarette smoking or water pipe and pipe smoking), duration and age at onset of use, and age at quitting. In this study, an overall tobacco smoker was one who had smoked cigarette, water pipe, or pipe for most days of the week over at least 6 months. One who had used to smoke cigarettes or water pipe in the past but quit and at the time of the study did not smoke was defined as an overall past smoker. Smokers who, at the time of the study, had the same pattern of use and consumption as the past were defined as current smokers. A non-smoker was someone who had not smoked cigarette or water pipe in the past, nor did s/he smoke at the time of the study [16].

Economic status was estimated by using principal component analysis (PCA) based on their assets, and participants in both phases were classified into three groups of high, medium, and low in terms of the economic status.

To examine the relationship between variables and the probability of transition in the stages of smoking from non-smokers to smokers, and past smokers, data were analyzed as panel longitudinal data. Panel data are those where variables are measured two or more times in different time periods. There are two types of data in the panel data: the data for each phase that show the differences between people and time series data which show the status of a trait in a person in the two phases of the study. The panel data regression allows to investigate both kinds of changes [17]. In this study, population-averaged logit model was used to examine the impact of independent variables on smoking in adults in the 5-year period. In this model, variables either changed during the two phases (time varying) such as age, economic status, and marital status or suggested as constant in the two phases (time invariant) such as gender, education level, and occupation. Outcome of the study was tobacco smoking, and tobacco use was added as a time-varying outcome to the model.

To estimate the prevalence of tobacco use by age and gender and smoking over the years, since changes in this age group were not big and were not affected by the 5-year period, the marginal model was used. In this model, the relationship between people in the two phases is not taken into account [18]. Here, the estimate of the age prevalence of smoking was a better estimation because age was not the same for people (time varying) and in addition, there was no heterogeneity in phase 1 and 2 data and it was possible to combine the data. Then, based on the observed prevalence, the smoothed prevalence graph of overall tobacco smoking and past tobacco smoking was drawn against the prevalence of non-smokers with multinomial P-spline method using the R software and simulation was used to estimate the confidence level of age-specific prevalence [19]. Considering the relationship between age and smoking, restricted cubic splines logistic regression model was used to estimate the probability of smoking at different ages and eventually the estimated probability for participants into three groups were coded and presented in graph. Data analysis was performed using STATA and R, and the significance level for all tests was 0.05. The effect of cluster sampling on calculating confidence limits for age- and sex-specific prevalence and on estimation of variable means was taken into account.

## Results

**Fig. 1:**
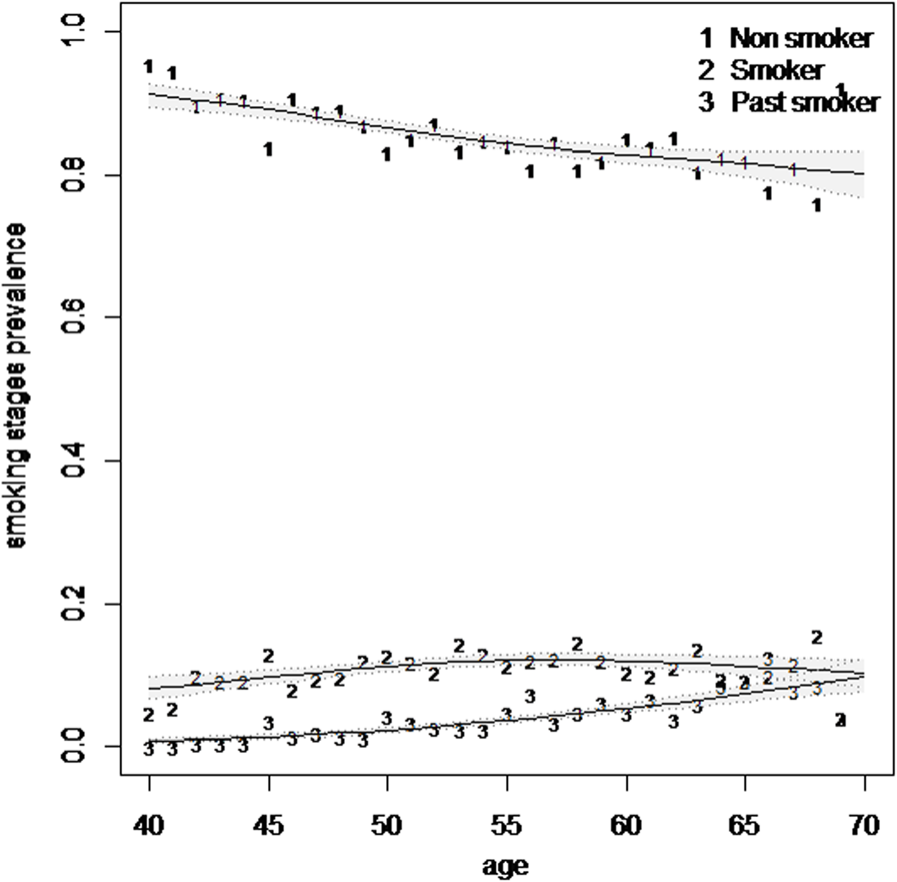
Smoothed prevalence of tobacco smoking stages in Shahroud population (40–69 years) using multinomial P-spline. The shaded areas represent 95% confidence intervals

**Fig. 2:**
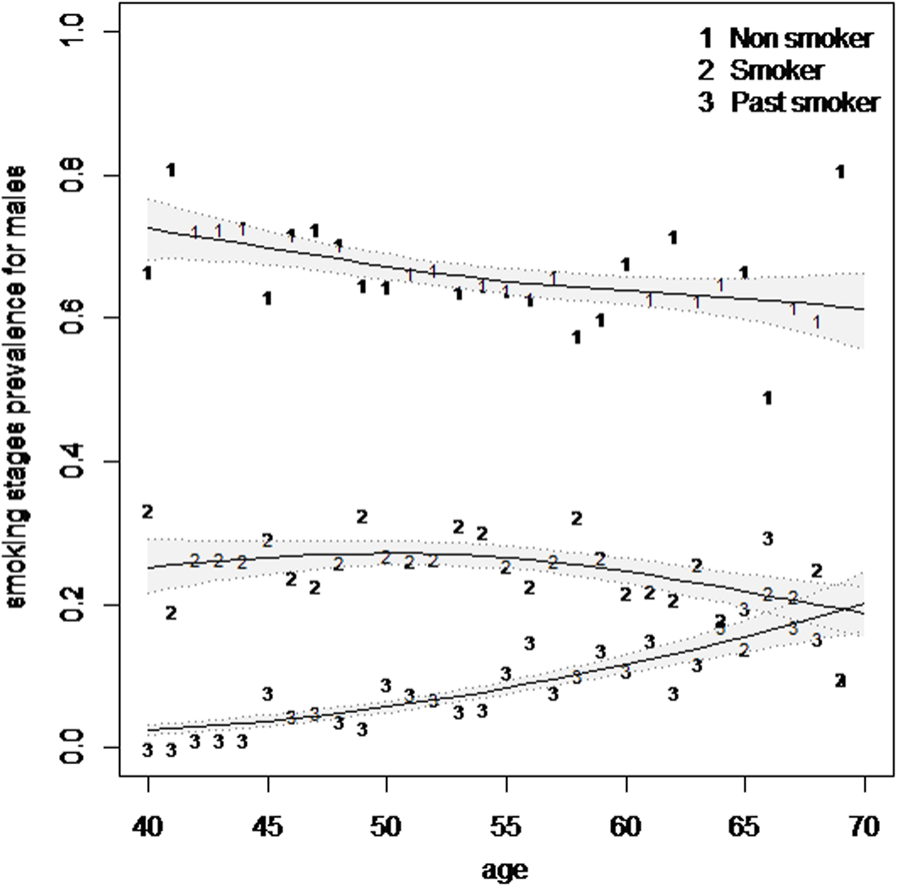
Smoothed prevalence of tobacco smoking stages in Shahroud male population (40–69 years) using a multinomial P-spline. The shaded areas represent 95% confidence intervals

In the second phase of the study in 2014, 4715 people out of 4737 participants were interviewed to determine their smoking status. On the whole, regardless of the period of the study, the data of 9902 participants on smoking status were analyzed to estimate the age- and sex-specific prevalence of tobacco smoking (cigarettes, water pipe, and pipe). The age- and sex-specific prevalence of overall tobacco smoking in this population is displayed in Table 1. The results of this table show that with the rising of age until the early 60s, prevalence of current tobacco smoking is also increasing. The age-smoothed prevalence of current tobacco smoking is shown in Fig. 1. With regard to the increase in overall past smokers, the increased prevalence of current tobacco smokers is not considerable (Fig. 1). With the age increasing in men, an increasing trend is observed in the prevalence of overall past smokers who do not currently smoke (Fig. 2).

**Table 1:** Age- and sex-specific prevalence of current and past smoking according to a marginal model in Shahroud, Iran (2009–2014)

Variables	No.	Prevalence (%)			
		Current cigarette smoking (95% CI)	Current water pipe smoking (95% CI)	Overall current tobacco smoking (95% CI)	Overall past smoker (95% CI)
Age group					
40–44	969	7.95 (6.3–10.0)	0.52 (0.2–1.2)	8.4(6.6–10.5)	0.31(0.1–0.95)
45–49	2247	9.66 (8.4–11.1)	0.71 (0.4–1.2)	10.2 (8.9–11.6)	1.6 (1.2–2.3)
50–54	2557	11.69 (10.5–13.0)	0.78 (0.5–1.2)	12.4 (11.2–13.7)	2.9 (2.3–3.6)
55–59	2130	11.41 (10.0–13.0)	1.03 (0.7–1.6)	12.4 (11.0–14.0)	5.2 (4.3–6.2)
60–64	1465	9.69 (8.2–11.5)	1.37 (0.9–2.1)	10.8 (9.2–12.6)	5.7 (4.8–6.9)
65–69	534	7.87 (5.9–10.4)	2.43 (1.4–4.1)	10.11 (7.8–13.0)	8.6 (6.5–11.3)
*χ*^2^ trend (*P* value)		0.43 (0.51)	16.5 (< 0.001)	4.9 (0.027)	128.6 (< 0.001)
Sex					
Female	5817	0.17 (0.09–0.3)	0.83 (0.6–1.1)	1.0 (0.8–1.3)	0.07 (0.03–0.18)
Male	4085	24.7 (22.9–26.7)	1.18 (0.9–1.6)	25.6 (23.7–27.6)	8.6 (7.7–9.6)
Total	9902	10.30 (9.5–11.2)	0.97 (0.8–1.2)	11.25 (10.4–12.2)	3.6 (3.2–4.0)

The results in Table 1 show that the prevalence of current tobacco smoking in the 40–69-years age group was 11.1% (95% CI 10.3–12.0) and the prevalence of current tobacco smoking was 1.0% (95% CI 0.8 to 1.3) among women and 25.6% (95% CI 23.7–27.6) among men. The prevalence of cigarette smoking was 24.7% among men and 0.17% among women, while the prevalence of water pipe smoking among women was 0.83%, the comparison of which with 1% prevalence of current tobacco smoking among women indicates that the majority of them were water pipe smokers. Moreover, the results in Table 1 showed that compared to women, the prevalence of water pipe smoking is higher among men (1.18%). The prevalence of overall past smokers in this population was 3.6% (95% CI 3.2–4.0) which was significantly higher in men than in women (8.6 versus 0.07%). Comparison of Table 1 and Figs. 1 and 2 shows an increasing trend in the prevalence of overall past smokers in this population.

**Fig. 3:**
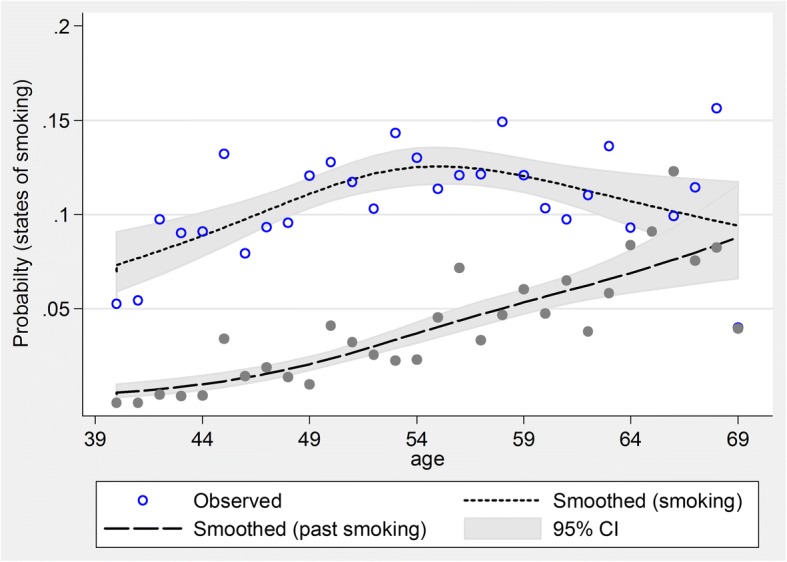
The estimated probability of current tobacco use and overall past smoking using a restricted cubic regression model in Shahroud, Iran

Considering the relationship observed between age and the smoothed prevalence of smoking and smoothed prevalence of past smoking in Figs. 1 and 2, a restricted cubic logistic regression model was used to examine the relationship between age and smoking, and using the fitted model, the probability of smoking and past smoking is shown in Fig. 3. As this figure shows, with an increase in age up to 55 years, the probability of smoking increases and thereafter the probability decreases and hence the trend of smoking quitting speeds up.

The average onset age of smoking is 25.64 years (95% CI 25.07–26.21), 25.63 years (95% CI 25.06–26.20) among men and 26.9 years (95% CI 20.3–33.5) among women. The average number of cigarettes smoked per day was 12.3 cigarettes (95% CI 11.5–13.0), and the maximum number of cigarettes was smoked by the 55–59-years age group with an average of 14 cigarettes smoked per day (95% CI 12.2–15.8). In this study, 20.7% of participants in the second phase of the study were passive smokers.

In the univariate analysis, the highest prevalence of tobacco smoking was among individuals with university degree of education (14.8%) and the lowest prevalence was among illiterate people with 6.8%. The prevalence of tobacco smoking in elementary school, junior high school, and high school were respectively 10.7%, 12.9%, and 11.8%. The relationship between education and tobacco smoking was significant (*P* < 0.05). Comparison of the prevalence of tobacco smoking based on marital status shows 5.3% for single people, 11.9% for married people, 3.4% for divorcees, and 10.4% for widows or widowers. The prevalence of tobacco smoking in terms of economic status did not show significant differences. The prevalence of tobacco smoking in high, medium, and low economic classes were respectively 11.4%, 11.7%, and 10.5%. In this study, there was a significant relationship between tobacco smoking and occupation, so that the highest prevalence was in the group of the unemployed (33.3%) and the lowest was among housewives (1.4%).

Since the data were longitudinal, population-averaged logit model was used to examine the relationship between age, gender, education, occupation, marital status, and economic status, and overall tobacco smoking. The results of this model are presented in Table 2. The final model results showed that male gender (OR = 53.9), average high economic status within study period (OR = 1.2), unemployed status (OR = 2.0), and marital status (widows and widowers (OR = 4.5) and divorces (OR = 3.3)) were associated with overall tobacco smoking in the population. In this model, the average age and education in the study period showed no relationship with overall tobacco smoking.

**Table 2:** Multiple population-averaged logistic regression for predictors of tobacco smoking among middle-aged population of Shahroud, Iran; 2009–2014

Variables		Odds ratio	95% CI	*P* value
Sex	Female	Reference		
	Male	53.9	28.3–102.9	< 0.001
Marital status	Single	Reference		
	Married	1.8	0.8–3.6	0.13
	Widow	4.5	1.8–11.0	< 0.001
	Divorced	3.3	1.0–10.4	0.045
Job	Employed	Reference		
	Retired	0.97	0.8–1.2	0.7
	Unemployment	2.0	1.2–3.3	0.009
	Disabled	1.0	0.5–2.1	0.92
	House keeper	1.5	0.8–2.7	0.21
	Others	1.6	1.3–1.9	< 0.001
Economic status	Low	Reference		
	Medium	1.2	0.98–1.3	0.08
	High	1.2	1.0–1.4	0.04

*CI* confidence intervals

Moreover, a population-averaged logistic regression model was performed to examine the relationship between the variables of education, marital status, economic status, age and gender, and being an overall past smoker. According to this model, age and gender showed relationship with increase in the odds of being an overall past smoker (1.1 per year of age rise and 5.2 for men compared to women, respectively) and other variables were not associated with increasing the odds of being an overall past smoker.

**Fig. 4:**
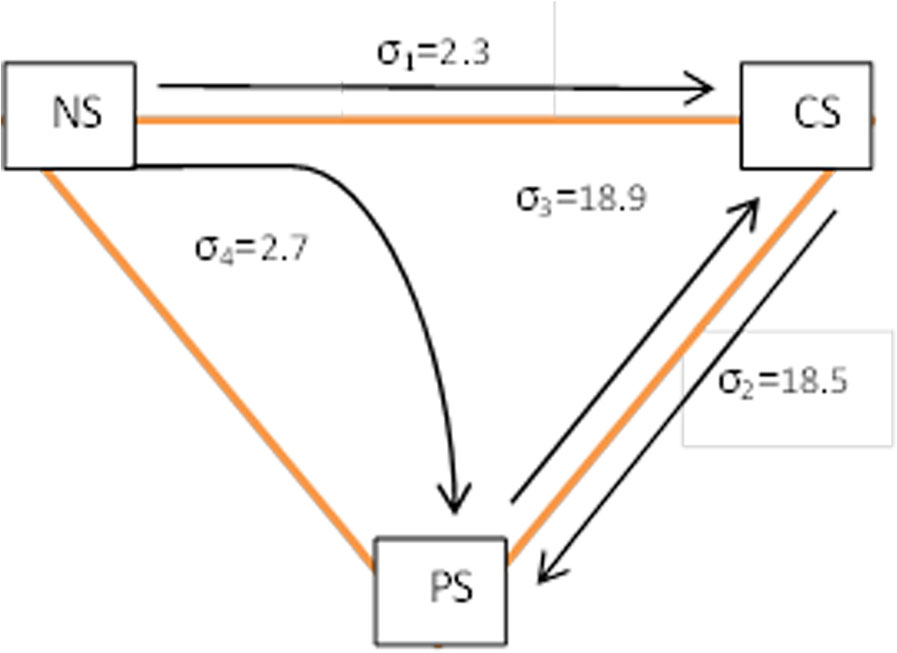
Probability of transition (%) in smoking stages during the 5-year period in the population of 40–69 years, Shahroud, Iran

Figure 4 shows the probability of transition between different stages of tobacco smoking where *σ*_1_ to *σ*_4_ indicate the percent of transition between stages (*σ*_1_ = transition from non smoker to current tobacco smoker, *σ*_2_ = transition from current smoker to past smoker, *σ*_3_ = transition from past smoker to current tobacco smoker, *σ*_4_ = transition from non smoker to past smoker) during the 5-year period. It was found that during this period of 5 years, the probability of transition of a non-smoker to an overall current tobacco smoker (*σ*_1_) was 2.3% (94 out of 4128 people during this 5 years transmitted from non-smokers to overall current tobacco smokers). Meanwhile, 18.5% of overall current tobacco smokers (95 out of 513 persons) had changed into past smokers (*σ*_2_) during the 5-year period. During the same period, 18.9% of past smokers (14 out of 74 people) started to smoke again (*σ*_3_).

## Discussion

As the results of this study indicate, the prevalence of cigarette smoking obtained through marginal model for the total of 2009 and 2014 was 10.3% (24.7% among men and 0.17% among women). In an article published based on the first phase of the study in 2009 [16], the prevalence of cigarette smoking was 10.8% (24.9% among men and 0.3% among women). Therefore, the results show that the prevalence of cigarette smoking in the two time points were similar and were not influenced by the time interval, and to have a better estimate of the prevalence of smoking in the population, it is possible to combine the data obtained in the two phases and present them as a marginal model which is in fact a kind of meta-analysis of individual data. By comparing the age-specific prevalence of smoking in other studies in Iran and age-specific prevalence of smoking in our study, it appears that the prevalence of cigarette smoking in other studies is higher than that in the present study [9, 10, 20]. Meysami et al. in their study showed that in the age group 35 to 65 years the prevalence of cigarette smoking varies between 13.4 and 19.9% [10]. One reason for this difference may be the low rate of cigarette smoking among the female population in Shahroud. Since smoking is a social stigma for women in Iran, it is usually understated in reports from traditional societies [10] and in a study which compared self-reported smoking and serum cotinine level as indicator of nicotine metabolites among women, a significant difference was observed between the two reports (1.3% vs. 6.7%) [21].

The results of other studies in various countries are presented in Table 3. By comparing the results of these studies, it appears that compared with other countries, the prevalence of tobacco smoking in this study is the lowest. Comparing the prevalence of tobacco smoking in Shahroud with the prevalence of tobacco smoking in 11 Mediterranean countries and North Africa and Pakistan [22], which are similar in terms of age groups, showed that the prevalence of tobacco smoking in both genders in our country is lower than that in these countries. In most studies of smoking, the ratio of women to men is 1:10 [2], while in our study, this ratio is 1:145 and for overall tobacco smoking, the ratio is 1:25. The difference between the prevalence of cigarette smoking and overall tobacco smoking is also due to the fact that most women in Iran are housewives (in this study, 87% of women were housewives) and because of the pattern of traditional societies, they use water pipe. Cigarette smoking is more a male pattern. However, in some traditional societies in Iran, water pipe smoking is also common among men [8]. Also, in many Middle Eastern countries, smoking is an indecent and shameful behavior for women, while in the same community, it is a normal behavior for men [22, 23]. Several studies have demonstrated that water pipe smoking is on the rise among adolescents and young adults in most countries and a study in Middle East reported the prevalence of water pipe smoking in the age group 13–15 years about 6 to 34% [24]. In Iran, the prevalence of water pipe smoking among high school students was estimated to be 6% (95% CI 5.1–6.9) [25].

**Table 3:** Comparison of the prevalence of smoking in various countries

Country	Year of study	Type of smoking	Age	Male	Female	Overall	Cigarette smoking cessation
Iran [10]	2007	Cigarette smoking	15–64	23.4	1.4	12.5	3.4
Iran [9]	2009	Cigarette smoking	15–64	22.1	1.3	11.8	1.7
Iran (present study)	2014	Cigarette smoking	40–70	24.7	0.17	10.3	3.7
Iran (present study)	2014	Tobacco smoking	40–70	25.6	1.0	11.25	3.6
Saudi Arabia [29]	2015	Tobacco smoking	> 15	21.5	1.1	12.2	–
Egypt [22]	2011	Tobacco smoking	≥ 40	53.5	1.8	29.6	–
Lebanon [22]	2011	Tobacco smoking	≥ 40	59.9	47.3	53.9	–
Morocco [22]	2011	Tobacco smoking	≥ 40	29.7	1.4	15.3	–
UAE [22]	2011	Tobacco smoking	≥ 40	37.4	8.6	23.5	–
Iran [7]	2009	Tobacco smoking	≥ 15	26.0	2.0	–	–
Albania [32]	2009	Tobacco smoking	≥ 15	42.5	4.2	–	–
Ukraine [32]	2007	Tobacco smoking	≥ 15	52.0	14.8	–	–
Iraq [7]	2009	Tobacco smoking	≥ 15	31.0	4.0	–	–
Italy [7]	2009	Tobacco smoking	≥ 15	33.0	19.0	–	–
Turkey [7]	2009	Tobacco smoking	≥ 15	47.0	15.0	–	–
USA [7]	2009	Tobacco smoking	≥ 15	33.0	23.0	–	–
UK [7]	2009	Tobacco smoking	≥ 15	25.0	23.0	–	–
Denmark [7]	2009	Tobacco smoking	≥ 15	30.0	28.0	–	–
Germany [7]	2009	Tobacco smoking	≥ 15	33.0	25.0	–	–

Although the age-specific prevalence of tobacco smoking increased up to nearly 55 years in this study, in later years, the overall tobacco smoking trend is declining. Meysami et al. reported a higher prevalence of tobacco smoking in the age group of 45 to 55 years compared to other age groups [10]. In a study by Fotouhi et al. on 3396 people older than 15 years in Tehran, the prevalence of smoking significantly increased from 3.4% in the age group 15–24 to 20.6% in 45–54 years group and then decreased again [9]. Results of other studies also show until the fifth and sixth decades of life smoking trend is on the rise [11, 16, 26] and then it is on the slump [11, 16] while the prevalence of past smokers increased in these years [9, 16]. Risk of non-communicable diseases and increased morbidity and mortality rate after age 55 can explain this downward trend [9]. In our study, age was not associated with tobacco smoking as a significant variable in a multiple regression model which is in agreement with the results of Khabab study [22]. This can be related to the age group under study, because as other studies indicate, smoking in the age groups of 35 to 55 years shows a more stable trend and later it declines [9]. Increased prevalence of smoking in age groups of 40 to 60 years in fact indicates a generation who started smoking 20 to 30 years ago at the beginning of the epidemics of smoking in the developing countries, when they were not aware of the harms of smoking and the laws prohibiting the use and sale of tobacco in public places was not implemented in the country either [16, 22].

The mean age for starting cigarette smoking was 25.64 years (among men, it was 25.63 years, and among women, it was 26.9 years), which is similar to the results of the study conducted in Tehran [9] and lower than the average age of starting smoking in Meysami study [10]. But since age groups in the two studies are different, a sound judgment may not be passed; however, the results of studies show decline in the onset age of smoking in Iran [27, 28]. Most people graduate from college at the age of 22 to 25, and they begin to apply for jobs and unemployment can be one of the causes of smoking in this age group [9]. Moreover, studies conducted with university students indicate that more than 54% have started smoking before entering the university [28].

In our study, the percentage of passive smoking was 20.7%, which is similar to the results of the study in the Saudi Arabia, where it was 23.1% [29], and in a similar study in Tehran, 16.5% of the participants were passive smokers [30].

In the multivariate data analysis model, despite the higher prevalence of smoking among educated people, there is no significant relationship between education level and tobacco smoking. In studies conducted in developing countries, a significant relationship has been observed between education and smoking [9, 23]. In the fitted model, the unemployed have a higher odds of smoking than the employed (OR = 2.0). Other studies have emphasized the negative effect of unemployment on overall tobacco smoking, and in some cases, smoking has been a way to reduce the stress of unemployment [16, 31]. Other studies have found that smoking is more prevalent among widows, widowers, and divorcees [11, 16]. In the present study, widows and widowers (OR = 4.5) and divorced (OR = 3.3) had higher odds of tobacco smoking compared with single people. Average high economic status within the time period of the study was associated with higher odds of smoking compared to the average low economic status groups, although the observed relationship is a borderline one. According to the WHO’s report, in high-income countries, the prevalence of overall tobacco smoking among women is higher than low-middle and low-income countries [7] and in another study which was conducted in several countries to identify the social factors and overall smoking, higher smoking among women in wealthier communities has been reported. As the abovementioned study reports, in countries such as Moldova, Ukraine, Honduras, Ghana, and the Dominican Republic, poorer men smoke more cigarettes than wealthier men, and in countries such as Jordan, the Dominican Republic, Moldova, Albania, Armenia and Bolivia, richer women smoke more cigarettes than poorer women [26].

As shown in Fig. 4, during the 5-year period of the study, only 2.3% of non-smokers changed into current tobacco smokers, while 2.7% during the same period started to smoke cigarette or water pipe and then gave up smoking (overall, a total of 5% of non-smokers changed into current smokers or past smokers), which indicates no significant change in the patterns of smoking between the two time points. Factors related with the pathogenesis of smoking in this transition between stages can also be studied in further research projects.

Given the main aim of the cohort study which was clinical and eye examination of the sample population, and noting the precision of measurement and the follow-up conducted throughout the study, it seems that there is the least bias in the data and subject selection in the study. In the second phase of the study, participants were electronically registered and the data of the first phase were also rechecked for errors in data entry. Using a sufficient sample size to estimate the prevalence and to fit the models is among the strengths of the current study. To improve the accuracy of estimating the prevalence of overall tobacco smoking, the meta-analysis of individual data in the two phases of Shahroud Eye Cohort Study was conducted, while considering the impact of the study design. However, due to limitations in the age group under study, it seems that the influence of age on increasing the chance of smoking cannot be shown well. Another limitation of the study can be the implementation of the study among the urban population.

## Conclusion

The results of this study show that in the urban population of Shahroud, the prevalence of overall tobacco smoking is higher among men and it is lower in comparison to other parts of Iran. When compared with the results of studies in the Middle East and other parts of the world, the prevalence of smoking in this population is low. Given the results of other studies, the age group of 35–55 has the highest prevalence in different societies, and this indicates that the estimated prevalence of smoking in this study can be the highest prevalence of smoking, which is again lower than other countries. The results show the increasing trend of past smokers in the population under study after the age of 55. Overall, 5% of the non-smokers have changed into current tobacco smokers or overall past smokers over the 5-year period. Given the relationship between unemployment, economic status, marital status, and gender and increase in the odds of smoking, education and awareness raising on the dangers and harms of smoking for the at-risk groups seem necessary. Collaboration between health care centers, media, and educational centers can help to raise public awareness and consequently help smokers to quit and help past smokers not to relapse into smoking.
